# 

**Published:** 1891-03-28

**Authors:** 


### BOOKS RECEIVED.

Hogg, John (13, Paternoster Row).
"Dr. Koch's Remedy—the Treatment of Consumption." A. E. Bridger, M D. Price 1s.

Lockwood, Crosby, and Son.

"Ventilating, Air Testing, etc." William Paton Buchan, R.P.

Macdougall, Alex. (Glasgow).

"Scheme for a Pathological Index." James C. Howden, M.D.

Paterson, William.

"What Ails the Baby?" John Dewar, M.D. ("Red Cross" Series.) Price 1s.

Stock, Elliott.

"On Consumption of the Lungs and Decline, and its Successful Treatment." George Thomas Congreve. Price 1s.

### NOTICE TO CORRESPONDENTS.

All MS., Letters, Books for Review, and other matters intended for the Editor should be addressed The Editor, The Lodge, Porchester Square, London, W.

The Editor cannot undertake to return rejected M.S., even when accompanied by stamped directed envelope.

**Notice to Advertisers and Subscribers.**

All Advertisements, Orders for Copies of The Hospital, and Business Communications should be addressed to The Publisher, *not to the Editor*, The Hospital, 140, Strand, W.C.

The Cost of Subscription, post free, is as follows:—Per week, 1½d.; per quarter, 1s. 8d.; per half-year, 3s. 3d.; per annum, 6s. 6d. Foreign:—Per annum, 8s. 8d.; payable, in all cases, in advance.

### Scraps and Gleanings.

The Munich police have forbidden the sale of Dr. Koch's lymph.

The Nottingham Hospital Saturday Committee collected during 1890 the sum of £1,559 8s.

The foundation stone of a hospital for infectious diseases, in North Brierley, was laid on the 14th inst.

The London County Council has placed a small gymnasium for children and young girls in the Victoria Park.

At the festival dinner of the Hospital for Sick Children, Great Ormond Street, subscriptions amounting to £4,360 were announced.

The present position of the Bolton Infirmary, as disclosed by the reports submitted at the annual meeting, is satisfactory in every respect.

On Saturday last Charles Lyddon was found "Not Guilty" of the murder of his step-brother, Dr. Reeks Lyddon, of which he had been accused.

Lord Rothschild presided at the festival dinner of the Jews' Hospital and Orphan Asylum. Subscriptions to the amount of £1,000 were announced.

It has been decided, at a public meeting in Runcorn, to erect a cottage hospital for that town. An institution of that kind is much needed in the locality.

An exhibition of collotype printing will be on view daily until April 4th, at 50, Great Russell Street, Bloomsbury, to which admission may be gained by presentation of visiting card.

The general meeting of the National Health Society was held on the 11th inst., when a most satisfactory report of work done during the past year was read and carried unanimously.

Mr. Alderman Davies has consented to preside at the anniversary festival of the British Orphan Asylum, Slough, at the Whitehall Rooms, Hotel Metropole, on Thursday, May 14th.

The Secretary of the Royal Hospital for Children and Women, Waterloo Bridge Road, have received subscriptions amounting to £152, in connection with the forthcoming annual dinner.

The scheme for the establishment of a cottage hospital at Wellington, which was originated by Mr. Edgerton Burnett, is receiving liberal support from other residents in the neighbourhood.

A Bill has been introduced into the Legislature of the State of New York to authorise the formation of a colony for epileptics. Provision is ultimately to be made for between 1,000 and 2,000 patients.

The Committee of the Great Northern Central Hospital, Holloway, have received a donation of £1,000 from the Goldsmiths' Company towards the £20,000 now being raised for the completion of the buildings.

The Committee of Management of the Richmond Hospital, after paying off all expenses for 1890, have a balance in hand of £248 19s., while the funded property has been increased by the sum of £102 11s. 4d.

A meeting, in connection with the Zenana and Medical Mission, was held at the Star and Garter Hotel, Richmond, on the 16th inst. The chair was taken by Lord Reay, and the Duchess of Teck was among those present.

At a meeting of the Court of Governors of Owens College, a committee was appointed to endeavour to raise by public subscription a sum of £50,000, which is required for the extension and increased cost of maintenance of the medical school.

An exhibition was held in the Inner Temple Hall last Saturday evening of work done by members of the Recreative Evening Schools Association. The work done in dressmaking, plain sewing, knitting, and crochet was most creditable, and showed a distinct advance on that exhibited last year.

The Duke and Duchess of Connaught have consented to patronise the concert on behalf of the Army Guilds' Home for the Orphan Daughters of Soldiers, to be given, by permission of the Duke of Westminster, at Grosvenor House, on Wednesday, April 29th, at three o'clock.

The Italian Government have appointed Dr. Angelo Mosso, Professor in the Royal University at Turin, to represent them at the forthcoming Congress of Hygiene and Demography in London next August. The Belgian Government have also announced their intention of appointing official delegates to the Congress.

The annual meeting of the subscribers to the Sheffield Children's Hospital was held on the 13th inst. The report stated that the out-patients numbered 3,[illegible]66, as against 3,2[illegible]4 in 1889, and the in-patients 181, as against 180. The total expenditure for 1890 was £1,126 14s. 9d., while the income was only £984 2s. 7d. The report was adopted.

In the report of St. John's Hospital for Diseases of the Skin, Leicester Square, reference is made to the fact that the hospital medical staff, having received a supply of Professor Koch's fluid, experimented with it, in the presence of 100 doctors, in the treatment of lupus patients. The results of these inoculations are said to have been satisfactory.

### THE STUDENT OF MEDICINE.

[All communications for this Supplement should be addressed to THE EDITOR of THE HOSPITAL, at 140, Strand, with the words, "Students' Column," written in the left hand top corner of the envelope.]

### NOTES FROM THE SCHOOLS.

### Oxford University.

The first meeting of the Junior Scientific Club of the present term, was held in the Physiological Laboratory at the Museum, and was very well attended. On the conclusion of the private business, the President called on Mr. R. T. Gunther, of Magdalen, for his exhibit of "Moller's Type Slide of Diatoms." Mr. Gunther said that in the slide he had to show there were 387 of these diatoms arranged in the space a quarter of an inch square; they were all mounted quite accurately in straight lines and right side up. Dr. Whitelock then read a paper on "Dr. Koch's Remedy for Tuberculosis." After giving a brief sketch of the history of consumption, he discussed the question of the contagiousness of phthisis, and next passed on to a consideration of micro-organisms producing disease in general, and of the tubercle bacillus in particular. He then gave the particulars of the line of research which led Koch to this discovery. Dr. Whitelock showed some of the fluid in question, which he stated was an extract of the attenuated bacilli. When the fluid was injected into a healthy subject it caused a slight sore and then healed up, but when tried in a person the subject of tuberculosis it caused violent inflammation of the diseased parts, and microscopic investigation showed that it had broken up the bacilli into innumerable pieces. The process of injection was then described, and the results of the injection on some patients he had seen in Berlin. He went to Berlin an enthusiast in Dr. Koch's treatment, but during his stay there he was gradually disillusioned, till he had come to the conclusion that the celebrated fluid was indeed a great success in cases of lupus, and one or two other local tubercular diseases, but in regard to consumption itself he had come to the conclusion that it was little use except in very early stages. After some remarks by Dr. Brooks, Dr. Collier, and Mr. Wilson, Dr. Whitelock replied, and the meeting adjourned.

Mr. A. W. Rucker, M.A., F.R.S., Professor of Physics at the Royal College of Science, London, formerly fellow, has been elected to an honorary fellowship at Brasenose College.

### Cambridge University.

DEGREES CONFERRED.—*Bachelors of Medicine and Surgery.*—Francis Wellford, Cuthbert Wyman, Trinity; Alfred Featherstone Kellett, William Wickham Simmons, St. John's; Harry Edward Smith, Gonville and Caius; Thomas Greenwood Crump, John Elsdale Molson, Emmanuel; Edward Spencer Blaker, Cavendish Hostel; Alfred Waugh Metcalfe, Trinity; Arthur Henry Bindloss, John Crossley Wright, St. John's; Victor Grey Molteno, Charles Rolfe, Clare; Thomas Horace Haydon, Charles Howard Usher, Gonville and Caius; Henry Sharland Pope, Cavendish Hostel.

### Middlesex Hospital.

A most successful smoking concert was held in connection with the Medical School, at the Portman Rooms, on the 18th inst. The chair was ably filled by Dr. Sidney Coupland, who was supported by Mr. Sydney Jones, Dr. Finlay, Mr. Pearce Gould, and others. The programme, which had been designed and drawn by Mr. P. W. Williams, was a wonderful work of art. On the top were some drawings of the school buildings, in the centre the portrait of the chairman, and all around various comic scenes from student life. The concert was opened by a march on the banjo band conducted by Mr. O. C. Sibley. A great feature of the evening was the reappearance of Mr. Guy Drury, formerly a student of the Hospital, but who is now "on the boards." He was enthusiastically received, and gave in his own peculiar style "With a Little," and "The Little Interjection Oh!" and afterwards, with "Boy Jones," an exceedingly clever topical song, entitled "Costermongers' Duet," written for the occasion by the latter gentleman. Mr. Jones also sang "What are the Wild Waves Saying" and "Just as I Thought," both written for the evening, and he well deserved the prolonged applause which followed his singing. Of the other comiques, Mr. Richard Blunt, who, like Mr. Drury, is a deserter from the ranks of Æsculapius to those of Thespius, gave three capital songs in good style. Mr. A. P. Birch scored a great success with a clever laughing song, "I couldn't help but Laugh." Dr. J. J. Pringle received an enthusiastic encore for his splendid rendering of "Queen of the Earth," and was equally successful in a duet with Mr. C. E. Burkinyoung, "The

### The Hospital Advertiser.

### THE STUDENT OF MEDICINE—(*continued*).

Fisherman." The latter also sang "Molly Bawn" in excellent style; and Mr. J. E. Coulson's "Come into the Garden, Maud," given with great taste and expression, was most thoroughly appreciated. Dr. C. E. Sheppard, who throughout the evening presided admirably at the piano, played a "Polonaise" of Chopin's in first-class style; and Mr. W. E. L. Horner scored with a capital rendering of Raff's ever-popular violin solo, "Cavatina." At the end of the evening Messrs. C. Bayley and O. C. Sibley splendidly played a banjo duet, and merited a better hearing, but, as is so often the case, the junior students considered it the correct thing to become somewhat noisy towards the end of the proceedings, thus spoiling the pleasure of others, as well as demonstrating their recent release from the school-room. The programme, which contained no less than twenty-seven items, was not concluded till just upon midnight, and the whole concert was a most successful one throughout.

#### Edinburgh University.

The motion for the Union debate on February 18th was that the "Students' Representative Council" does not possess the confidence of the students. The arguments in support of the motion were mostly humorous rather than serious. There were several amendments, the principal one, that the S.R.C. had greatly benefited the students, and was worthy of their confidence. Protests having been raised against the divisions taken, and the meeting adjourned, it is difficult to say what was the result.

A protest has been issued for students to sign against the admission of lady students to the Royal Infirmary, on the grounds that the material available is not sufficient, and that their admission would interfere with the present clinique. It does not seem to have received much support. In one ward a counter-petition was raised.

The annual conference of the Students' Representative Councils of the Scottish Universities was held in the University Court Room on February 20th. The establishment of an inter-university annual, subject of cap and gown for students, and abolition of Saturday examinations, were discussed. The Song Book Committee announced that it would soon be ready. The delegates were entertained at dinner, and next day were shown round the University.

The ten-mile championship of the Hare and Hounds Club, held on February 20th, was won by G. W. Pollard, J. G. Ross being second. The club held an inter-club run with the Edinburgh Harriers from Duddingston, on February 28th.

The University Musical Society held its twenty-third annual concert on March 7th, which was a very great success. Miss Duncan's song, "Ave Maria," with organ and 'cello accompaniments by Dr. Greig and Herr Gallrein, was the event of the evening. This, and Dr. G. S. Meadows's song, were encored. Herr Alfred Gallrein's 'cello solos and Mr. F. L. Ehrke's violin solos were very loudly applauded. The orchestra played: Overture "Couronne d'Or," by Herman; Gounod's overture, "Mirella"; and Dr. J. Greig's march, "Orpheus."

The Union Government Debate was held on March 11th. There were many speakers on both sides, but Mr. Charles Stewart, for the Government, and Mr. D. B. Bogle, for the Opposition, were the best. The motion that the present Government is unworthy of the confidence of the country was lost by 60 to 28 votes.

The University Athletic Club held a special meeting to consider the advisability of acquiring a new field near to Edinburgh. To get to the present field it is necessary to go by train, and the service is very scanty and unpunctual. The suggested field is within fifteen minutes' walk of the students' quarters, and is in every way suitable. The great difficulty is the raising of funds. A committee was appointed to set about collecting funds.

### STUDENTS' MEDICAL SOCIETIES.

#### Royal Veterinary College Medical Association.

The 143rd ordinary general meeting of this association was held in the theatre of the college on Wednesday, February 11th, 1891, Mr. J. H. Manton, vice-president, in the chair. There were present the president, thirty student members, two visitors, and the assistant secretary. After the usual preliminary business, Mr. M. T. Sadler read a paper on "Veterinary Obstetrics." The essayist first mentioned the various causes of sterility in the domesticated animals. The changes which the uterus undergoes during the period of utero-gestation were then considered, together with the position of that organ before and after impregnation and the physical signs of this condition. The hygienic management of pregnant animals was fully considered, and the

THE STUDENT OF MEDICINE—(*continued*).

inadvisability of administering medicine to animals in this condition was referred to. The essayist concluded his paper by calling attention to accidents during pregnancy, viz., hernia of the uterus, rupture of the uterus, and abortion. Messrs. Evans, Garnet, Shepherd, Sessions, Martin, H. C. Taylor, and others entered in the discussion which followed, at the conclusion of which a hearty vote of thanks to the essayist for his paper, and to the Chairman for presiding, brought the meeting to a close.

### APPOINTMENTS.

At the Hospital for Consumption and Diseases of the Chest, Brompton, P. R. Dodwell, L.R.C.P. Lond., M.R.C.S. Eng., and E. L. Rouse, have been appointed house physicians. At St. Thomas's Hospital, Henry Hugh Clutton, M.A., M.B. Cantab., F.R.C.S. Eng., has been appointed surgeon. At St. John's Hospital for Diseases of the Skin, C. H. Evans, L.R.C.P., M.R.C.S., has been appointed house surgeon. At the St. Marylebone Infirmary, Notting Hill, Fred. Faichnie, M.R.C.S., L.R.C.P. Lond., has been appointed clinical assistant. At Bethlem Royal Hospital, Harry Corner, M.B., L.R.C.P. Lond., M.R.C.S., has been appointed second assistant medical officer. At the Cheltenham General Hospital, Doyd Cardew, M.R.C.S., L.R.C.P., has been appointed junior house surgeon. At the Brixton, Streatham, and Herne Hill Dispensary, H. M. Horden, M.B., L.R.C.P., has been appointed resident house surgeon. At the Grosvener Hospital for Women and Children, Charles Arthur Morris, M.A., M.B., Cantab, F.R.C.S., has been appointed surgeon. At the Liverpool Infirmary for Children, Myrtle Street, Maxwell Ogilvy-Ramsay, M.A., M.B., C.M.Edin., has been appointed house surgeon. At the Royal Edinburgh Hospital, H. G. Huie, M.B., C.M.Edin., and R. D. Rudolf, M.B., C.M.Edin., have been appointed resident physicians. At the Doncaster Infirmary, P. B. Mackay, L.R.C.P. Lond., M.R.C.S., has been appointed honorary surgeon. At the Bristol Hospital for Sick Children and Women, E. Leonard Lees, M.D., C.M., M.R.C.S., has been appointed medical registrar. At the Hope Hospital, Ernest Albert Shaw, B.A., M.B., B.C.Cantab., has been appointed assistant medical officer. At the Eye, Ear, and Throat Infirmary, Edinburgh, Alex. Black, M.B., F.R.C.P.Edin., has been appointed surgeon. At the French Benevolent Society, T. A. Gartley, L.D.S.R.C.S.Eng, has been appointed honorary dental surgeon. At the Royal Infirmary, Dundee, John Norman MacLeod, M.A., M.B., C.M.Glasg., has been appointed house surgeon. At Owens College, Manchester, Edwin Goodall, M.D., has been appointed demonstrator in pathology.

### VACANCIES.

The following vacancies are announced this week, the amount of salary in each case being given after the name of the institution:

**Resident Medical Officers.**

Bethlem Hospital, Two Resident Clinical Assistants.
Bridgewater Infirmary, House Surgeon—Salary £80.
County Asylum, Shrewsbury, Junior Assistant Medical Officer—Salary £100.
Chelsea Hospital for Women, Fulham Road, S.W., Resident Medical Officer—Salary £80, &c.
County Asylum, Rainhill, near Liverpool, Assistant Medical Officers—Salary £100, &c.
Deaconesses' Institution and Hospital, Tottenham, Resident House Surgeon—Salary £90, &c.
District Infirmary, Ashton-under-Lyne, House Surgeon—Salary £90, &c.
East Suffolk and Ipswich Hospital, House Surgeon—Salary £100, &c.
Hospital, King's Lynn, House Surgeon and Secretary—Salary £80, &c.
Hereford County and City Lunatic Asylum, Hereford, Assistant Medical Officer—Salary Two Guineas per week.
Hulme Dispensary, Manchester, House Surgeon.
Jaffray Suburban Branch of the General Hospital, Gravelly Hill, near Birmingham, Resident Medical Officer—Salary £150, &c.
Kent County Asylum, Chartham, near Canterbury, Junior Assistant Medical Officer—Salary £125, &c.
Liverpool Dispensaries, Assistant Surgeon—Salary £80, &c.
Metropolitan Hospital, Kingsland Road, N.E., House Physician—Salary £60, &c.; House Surgeon—Salary £60; Assistant House Surgeon.
Paddington Infirmary, Harrow Road, W., Resident Clinical Assistant—Honorarium.
Royal Hospital for Women and Children, Waterloo Bridge Road, S.E., Resident Medical Officer—Salary £70, &c.
Royal Bath Hospital and Rawson Convalescent Home, Harrogate, Resident Secretary and Dispenser—Salary £80, &c.
St. Marylebone General Dispensary, Welbeck Street, W., Resident Medical Officer—Salary £105, &c.
St. Thomas's Hospital, S.E., Resident Assistant Physician.
Stockport Infirmary, Assistant House Surgeon.
Worcester County and City Lunatic Asylum, Third Assistant Medical Officer—Salary £100, &c.

**Non-Resident Medical Officers.**

Cancer Hospital (Free), Fulham Road, S.W., Assistant Surgeon.
Charing Cross Hospital, Surgical Registrar—Salary £40.

### THE STUDENT OF MEDICINE—(continued).

Chelsea Hospital for Women, Fulham Road, S.W., Clinical Assistant.
Charing Cross Hospital Medical School, Demonstratership of Physiology —Salary £100.
Guy's Hospital Dental School, Two Anæsthetists.
Hospital for Women, Soho Square, W., Clinical Assistants.
King's College. London, Demonstratorship of Physiology.
Newcastle-on-Tyne Dispensary, Visiting Medical Assistant—Salary £120.
Norfolk and Norwich Hospital, Norwich, Physician.
Royal South London Dispensary, Surgeon.
Royal Hospital for Women and Children, Waterloo Bridge Road, S.E., Anæsthetist and Registrar.
St. George's and St. James's Dispensary, King Street, Regent Street, W., Surgeon.
St. Mary's Hospital, Paddington, W., Surgeon in charge of out-patients.
West London Hospital, Hammersmith Road, W., Assistant Surgeon.

### PASS LISTS.

#### Society of Apothecaries of London.

The following candidates have passed in surgery: A. Allen, Charing Cross Hospital; H. Collier, Liverpool University College and Middlesex Hospital; C. E. Cornwall, St. Bartholomew's Hospital; R. S. Freeland, L.R.C.P., M.R.C.S., Guy's Hospital: A. G. Haydon, St. Bartholomew's Hospital; C. G. Hutchinson, L K.Q.C.P.I., Birmingham Queen's College and Dublin; G. Jones, M.A., Oxford and London Hospital; W. F. H. Newbery, M.D., Trinity Medical College, Toronto; J. H. Roberts, Guy's Hospital; C. B. P. Spencer, Guy's Hospital; R. D. Wagborn, Westminster Hospital; F. C. Wood, L.S.A., London Hospital.

The following candidates passed in the subjects indicated:—

Medicine, Forensic Medicine, and Midwifery.—T. S. Byass, University College; H. Fairfax, Charing Cross Hospital; F. Lewis, St. Mary's Hospital; F. H. Lowe, St. Bartholomew's Hospital; H. F. S. Nunes, St. Mary's Hospital; C. B. P. Spencer, Guy's Hospital; J. F. Twist, Queen's College, Birmingham.

Medicine and Forensic Medicine.—W. R. Thomas, M.D., McGill, Montreal, and London Hospital.

Medicine and Midwifery.—W. R Willey, St. Mary's Hospital.

Forensic Medicine.—W. R. Nicol, M.D., McGill, Montreal; W. E. Toyne, Sheffield and Edinburgh.

The following have received the Diploma of the Society, having passed in all the subjects required for registration: A. Allen, T. S. Byass, C. E. Cornwall, H. Fairfax, F. Lewis, F. H. Lowe, H. F. S. Nunes, C. B. P. Spencer, J. F. Twist.

#### Examining Board in England by the Royal Colleges of Physicians and Surgeons.

The following gentlemen, having passed the necessary examinations, have been admitted Diplomates in Public Health: Joao Francisco Braga, L.S.A., King's College and Edinburgh; William Haig Brodie, M.D. Edin., Edinburgh University; Henry Cropley, F.R.C.S. Eng., Lond[illegible] Hospital; Sidney Reginald Dyer, M.D. Brux, L.R.C.P. Lond., M.R.C.S[illegible] Middlesex Hospital; Oliver Field, M.D. Edin., L.R.C.P. Lon[illegible] M.R.C.S., Edinburgh University; Robert Hammel Firth, F.R.C[illegible] Eng., University College Hospital; George Henry Ha[illegible] M.R.C.S., L.R.C.P. Edin., Birmingham; George Ernest Hasl[illegible] M.D. Brux., L.R.C.P. Lond., M.R.C.S, London Hospita[illegible] Frank Hicbens, M.D.Lond., M.R.C.S., London Hospital; John Holroy[illegible] M.R.C.S London Hospital; Edward Hussey, M.D. Durh., L.R.C.P. [illegible] S. Edin., Sheffield, Edinburgh, and Charing Cross Hospitals; Ha[illegible] Richard Kenwood, M.B. Edin., L.R.C.P. Lond., London Hospita[illegible] Frederic William Dobson McGachen, L.F.P.S. Glasg., London Hospit[illegible] Clement Mallins, M.D. Dub, Dublin; John Norton, M.D. Dur[illegible] M.R.C.S., Westminster Hospital; Walter Roughton, L.R.C.P. Lon[illegible] M.R.C.S., St. Bartholomew's Hospital; Alexander Williamson Stirli[illegible] M.D. Edin., Edinburgh and University College Hospital; William Sc[illegible] Tebb, M.D. Camb., L.R.C.P. Lond., M.R.C.S., St Thomas's Hos[illegible] and Cambridge; Leonard Wilde, M.D. Durh., L.R.C.P. Lond., M.R.C[illegible] St. Thomas's Hospital.

#### Royal College of Surgeons of England.

The following gentleman, having previously passed the necessary examinations, and having now attained the legal age (twenty-five years) was, at the ordinary meeting of the Council on the 12th inst., admitted a Fellow of the College: Arnold Caddy, L.R.C.P. Lond., of St. George's Hospital. Diploma of Member dated August 4th, 1887.

### ATHLETICS.

#### Football.

##### Rugby.

Inter Hospital Challenge Cup (Semi final tie).—On Wednesday, the 18th, a large crowd collected on the Richmond Athletic Association Ground to witness the match between Middlesex and Guy's. Middlesex kicked off against the wind, but Guy's with the wind on their side pressed hard, and several times looked like scoring. The Middlesex forwards, however, played well, so that at half-time neither side had made a point. At the beginning of the second half of the game Middlesex appeared to be having much the best of it, Williams and Lee both playing very well and markedly distinguishing themselves. When the whistle finally sounded neither side, however, had scored a point, [illegible] the game resulted in a draw. It will be remembered that the original draw was Thomas v. Middlesex and Guy's a bye. The match between Middlesex and Thomas a fortnight ago also ended in a drawn game. [illegible] new draw then was obtained, with the result that Thomas drew the bye and Middlesex played Guy's. It was at one time generally reported that since this last draw Thomas had been disqualified, having played a man out of his year, but this is not as yet quite settled.

